# Glucose starvation triggers filamentous septin assemblies in an *S. pombe* septin-2 deletion mutant

**DOI:** 10.1242/bio.037622

**Published:** 2019-01-15

**Authors:** Minghua Liu, Maria B. Heimlicher, Mirjam Bächler, Chieze C. Ibeneche-Nnewihe, Ernst-Ludwig Florin, Damian Brunner, Andreas Hoenger

**Affiliations:** 1 University of Colorado at Boulder, Department of Molecular, Cellular and Developmental Biology, UCB-0347, Boulder, CO 80309, USA; 2 University of Zürich, Department of Molecular Life Sciences, Winterthurerstrasse 190, 8057 Zürich, Switzerland; 3 University of Texas at Austin, Center for Nonlinear Dynamics and Department of Physics, Austin, TX 78712, USA

**Keywords:** Conventional and cryo-electron microscopy, Vitrified sectioning, Correlative light and electron microscopy, Septins, Filamentous septin assemblies, Glucose starvation in *S*. *pombe*

## Abstract

Using correlative light and electron microscopy (CLEM), we studied the intracellular organization by of glucose-starved fission yeast cells (*Schizosaccharomyces pombe*) with regards to the localization of septin proteins throughout the cytoplasm. Thereby, we found that for cells carrying a deletion of the gene encoding septin-2 (spn2Δ), starvation causes a GFP-tagged version of septin-3 (spn3-GFP) and family members, to assemble into a single, prominent filamentous structure. It was previously shown that during exponential growth, spn2Δ cells form septin-3 polymers. However, the polymers we observed during exponential growth are different from the spn3p-GFP structure we observed in starved cells. Using CLEM, in combination with anti-GFP immunolabeling on plastic-sections, we could assign spn3p-GFP to the filaments we have found in EM pictures. Besides septin-3, these filamentous assemblies most likely also contain septin-1 as an RFP-tagged version of this protein forms a very similar structure in starved spn2Δ cells. Our data correlate phase-contrast and fluorescence microscopy with electron micrographs of plastic-embedded cells, and further on with detailed views of tomographic 3D reconstructions. Cryo-electron microscopy of spn2Δ cells in vitrified sections revealed a very distinct overall morphology of the spn3p-GFP assembly. The fine-structured, regular density pattern suggests the presence of assembled septin-3 filaments that are clearly different from F-actin bundles. Furthermore, we found that starvation causes substantial mitochondria fission, together with massive decoration of their outer membrane by ribosomes.

## Abstract (precis)

**Summary:** We present a correlative electron tomography and light microscopy study on yeast cells under glucose starvation. We visualized several novel effects such as septin bundling and mitochondrial fission followed by ribosome decoration.

## INTRODUCTION

Most cellular systems and organisms have an emergency plan that allows them to adapt their metabolism to stressful situations such as nutrient starvation. When yeast cells run out of glucose (reviewed in De Virgilio, 2012) or nitrogen (Su et al., 1996), they enter a quiescent state, which allows them to survive for some time until nutrients become available again. Thereby, a key goal is to reduce energy consumption while preserving a certain level of cellular organization and maintenance. It has been shown previously that cells entering quiescence reorganize their cytoplasm. A number of publications have analyzed this reorganization for *Saccharomyces cerevisiae* (*S. cerevisiae*, or budding yeast; e.g. see De Virgilio, 2012, Rødkaer and Faergeman, 2014). However, for the fission yeast *Schizosaccharomyces pombe* (*S. pombe*), much less is known about the processes accompanying starvation. Besides arresting growth and its associated functions such as membrane trafficking, fission yeast cells also lose all known signs of cell polarization (Makushok et al., 2016). Similar to *S. cerevisiae*, actin in *S. pombe* reorganizes first into a number of globular assemblies that randomly move through the cells (Sajiki et al., 2009). In contrast, microtubule based traffic continuously slows down and microtubules eventually seem to disappear or cluster into a small hyper-stable bundle (Laporte et al., 2013, 2015). How these changes in cytoskeleton organization affect the position and organization of other cellular structures, such as the Golgi, mitochondria or the endoplasmic reticulum, is not known.

**Fig. 1.**
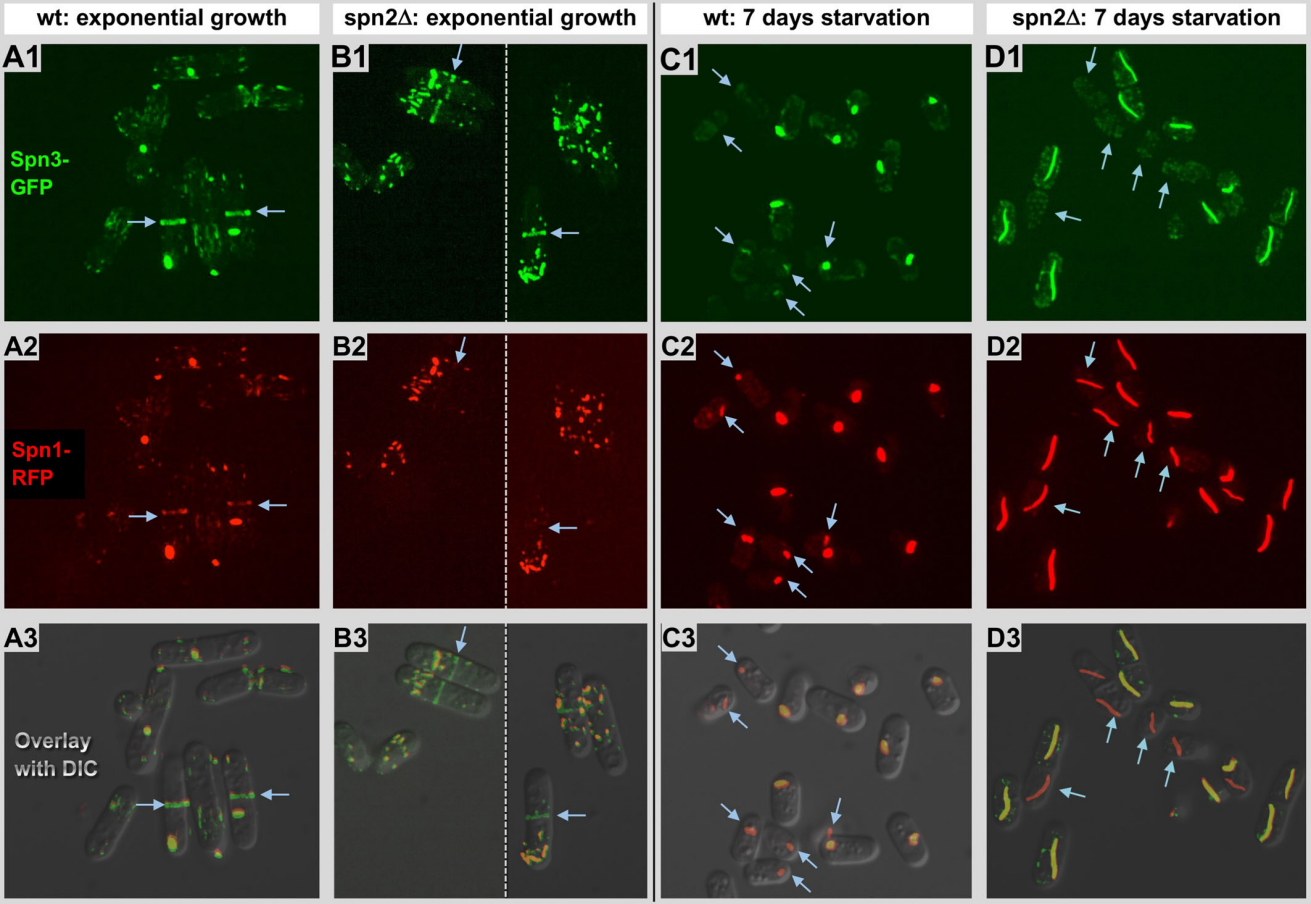
**Spn1p-RFP and spn3p-GFP expression and localization patterns.** (A1–A3) Exponentially growing cells expressing spn3p-GFP (A1, green), spn1p-RFP (A2, red), overlaid with a DIC image in A3. Both proteins can be found together, evenly distributed throughout the entire cytosol forming little clusters and accumulated at the periphery of septa in dividing cells (arrows; also see Fig. 4B). (B1–B3) Exponentially growing spn2Δ cells expressing spn3p-GFP (B1) and spn1p-RFP (B2). The panels are merges of two different images indicated by the dotted line. Both proteins assemble into globular clusters (also see Fig. 5A) or short filamentous assemblies. Spn3p-GFP can be found on septa while spn1p-RFP seems absent, or only present at a very low concentration [also see overlay (B3) and arrows]. (C1–C3) Starved cells expressing spn3p-GFP (C1) and spn1p-RFP (C2), after 7 days of culturing in low glucose medium (LMM). Both proteins aggregate and merge together into one single clump per cell, except for minor traces of protein that remain distributed throughout the cytosol. Evident from the overlay panel; not all clusters contain both proteins (arrows). (D1–D3) Starved spn2Δ cells expressing spn3p-GFP (D1) and spn1p-RFP (D2) after 7 days of culturing in LMM. Both proteins form prominent elongated filamentous structures, typically only one per cell. The large elongated assemblies in each cell all seem to contain spn1p-RFP or both, but interestingly, some cells lack the spn3p-GFP component (arrows; see overlay D3).

To better describe sub-cellular organization of starved *S. pombe* cells*,* we have analyzed the localization of various proteins in wild-type cells and in cells carrying genetic modifications. Amongst these were cells with deletions of septin genes including septin-2 deletion (spn2Δ), which is the main subject of this study. Septins are conserved GTP-binding proteins that associate with cellular membranes as well as the actin and microtubule cytoskeletons (Spiliotis and Nelson, 2006). They were first discovered in *S. cerevisiae* where they form a collar ring at the bud neck (Hartwell, 1971; Byers and Goetsch, 1976). It is believed that this septin collar provides a physical barrier for proteins and RNAs and serves as a scaffold for the recruitment of other proteins (Weirich et al., 2008). Septins localize throughout the cytoplasm in non-dividing cells (Fares et al., 1995; Spiliotis and Nelson, 2006; also see Fig. 1). They are involved in multiple processes including cell morphogenesis, membrane shaping and cytoskeleton dynamics (Hall et al., 2009). A recent study in human cells also demonstrated that septins build a cage-like structure to entrap intracytosolic bacteria (Mostowy et al., 2010). Septins have been linked to several human diseases such as neurological disorders and oncogenesis (Hall and Russell, 2004; Roeseler et al., 2009). They may form various polymers that assemble into filamentous structures forming meshworks, fibers or rings (An et al., 2004; Weirich et al., 2008). In *S. pombe*, septins are not essential (Versele and Thorner, 2005). Septin-1 (spn1p), septin-2 (spn2p), septin-3 (spn3p) and septin-4 (spn4p) are all expressed in vegetatively growing cells where they form hetero-octamer septin rods, which can further assemble into larger septin filaments (An et al., 2004; Hall and Russell, 2004). In exponentially growing cells performing cytokinesis, septins 1–4 form a ring structure in the cell center. The septin ring contributes to the assembly of the contractile actin/myosin-II ring that constricts to separate the cytoplasm of the two daughter cells similar to mammalian cells (Wu et al., 2010). Thereby, spn1p and spn4p are present during septin ring formation, while either spn2p or spn3p, although required for proper septum function, are often absent. However, there is no indication that any septins are required for ring formation (Wu et al., 2010). Septin-5 (spn5p), septin-6 (spn6p) and septin-7 (spn7p) are only expressed during meiosis and sporulation (Hartwell, 1971; Bähler et al., 1998; Onishi et al., 2010). X-ray crystal structures of septin polymers at near-atomic resolution are now available from *S. cerevisiae* (Bertin et al., 2010) and mammals (Sirajuddin et al., 2007).

**Fig. 2.**
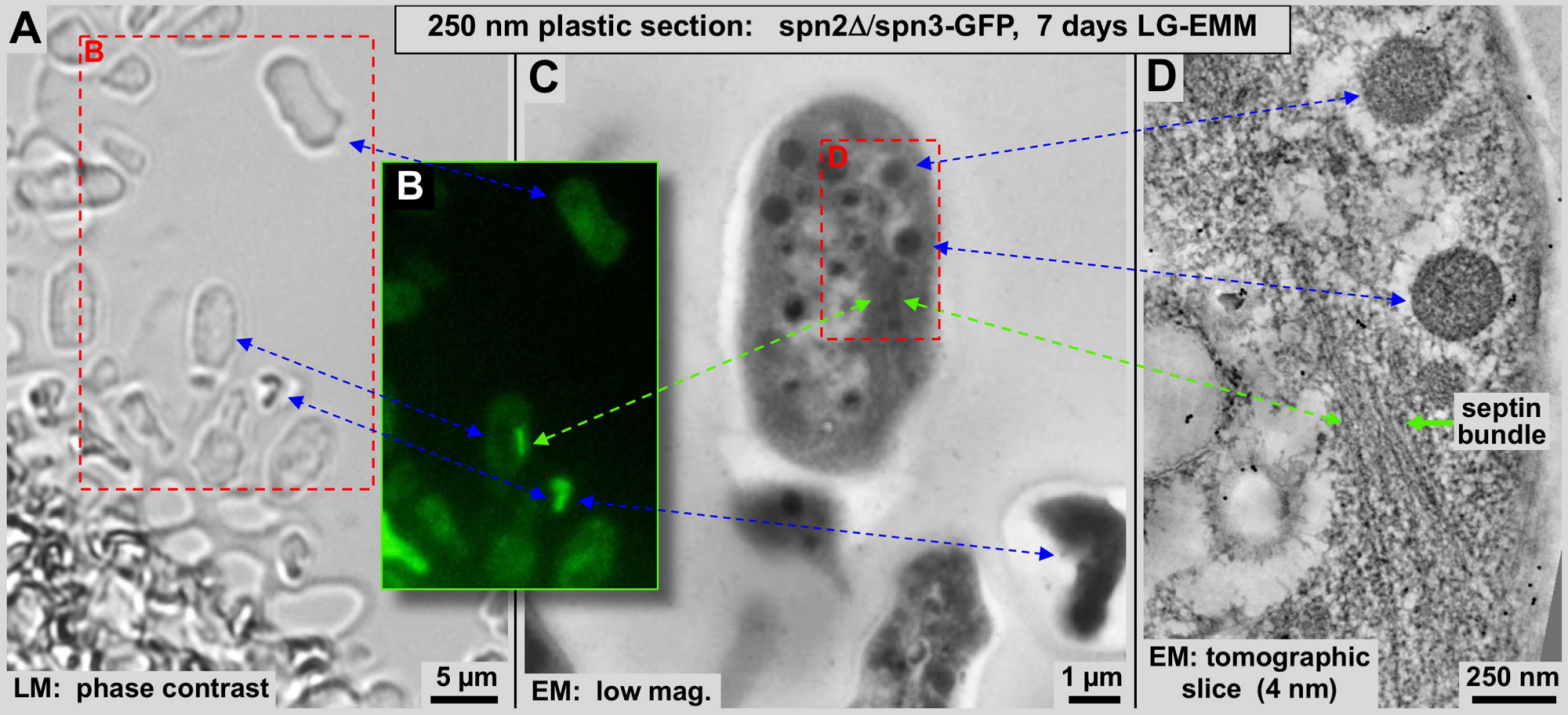
**Correlative light and electron microscopy performed on spn2Δ/spn3-GFP cells.** Arrows connect identical features that we can identify in the phase contrast image, fluorescence image and tomographic reconstruction of starved cells that have formed filamentous spn3p-GFP assemblies after 7 days of culturing LMM. A 250 nm thick plastic section of high-pressure-frozen, lowicryl-K4M embedded cells were mounted on an EM grid. An identical region on the grid was imaged on the grid by phase contrast (A: LM), by fluorescence light microscopy (B) and, after transfer to a 300 kV Tecnai-F30, with low-magnification as micrograph (C: EM) and a thin (4.0 nm) computational section through an electron tomogram (D). Green arrows connect the sites of the spn3p-GFP assemblies. Blue arrows connect other easily recognizable common features of the different panels such as high-density polymers, granules and entire cells. The red frame in A corresponds to the image area shown in B. The red frame in C corresponds to the image area shown in D.

**Fig. 3.**
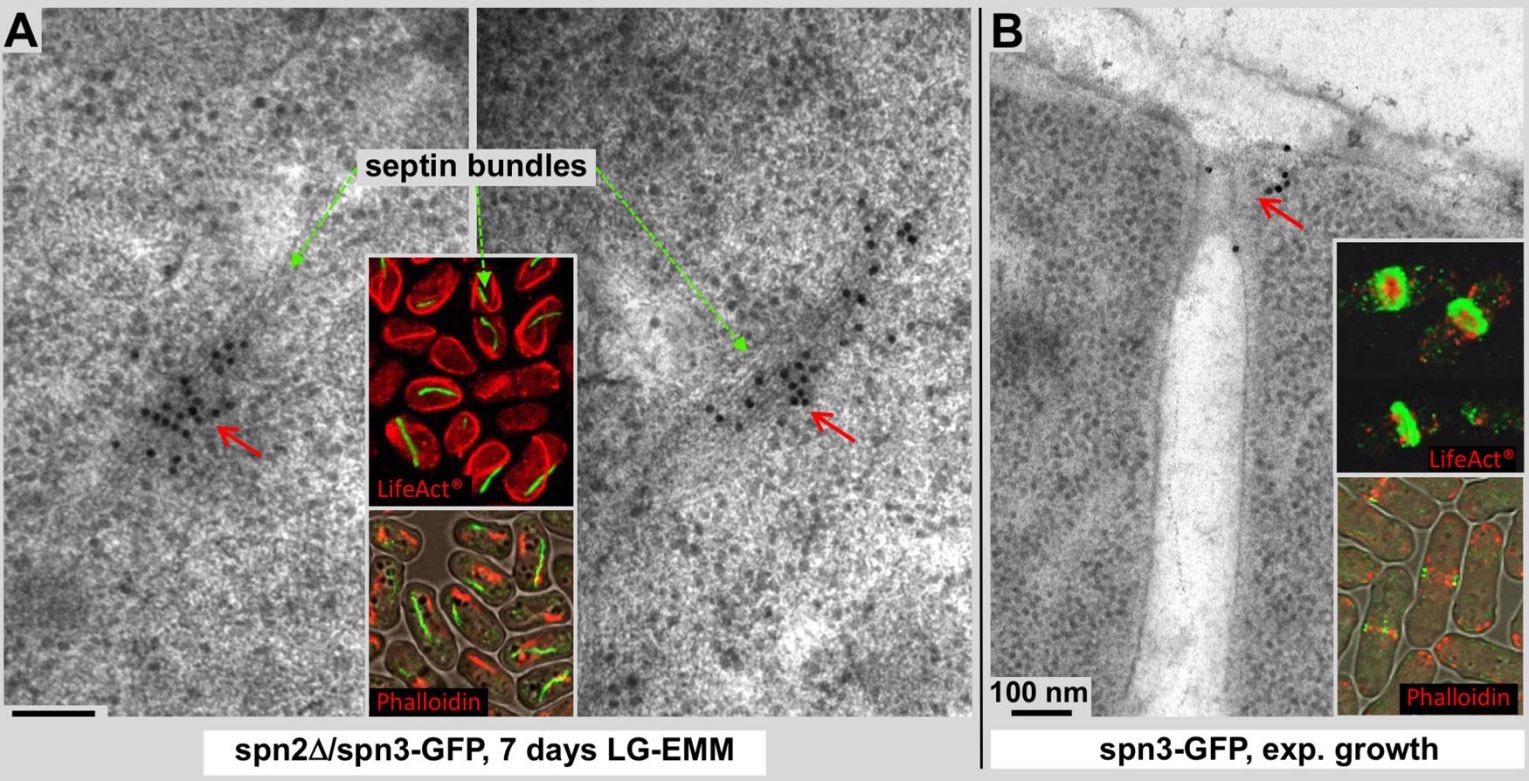
**Immunogold labeling on thin sections of spn2Δ/spn3-GFP and spn3-GFP cells.** Labeling was achieved with an anti-GFP primary antibody and a secondary antibody conjugated to 15 nm gold particles (arrows). (A) Immunolabeling provides another mode of correlation between the fluorescence signals (inset panel) and EM density data and confirms the presence of spn3p-GFP in the filamentous structures observed in starved spn2Δ cells after 7 days of culturing in low glucose medium (red arrows in both panels). Green dashed arrows indicate comparable septin bundles on electron micrograph and fluorescence images. The inset panels in A and B show spn3p-GFP fluorescence images of the corresponding cells in green, and actin staining in red (upper panels, LifeAct^®^-mCherry; lower panels, Rhodamine-Phalloidin). The lower insets display actin staining with Rhodamine-Phalloidin, overlaid with a phase-contrast image, to test for potential differences with LifeAct^®^-mCherry (also see Fig. 4). (B) The anti-GFP primary antibody binds to spn3p-GFP at the outer ring formed by the septa and the old cell wall in dividing wild-type cells during exponential growth (red arrow). The inset panels show dividing cells in a projection of a thin confocal slice (lower insets) and an entire confocal 3D image stack that is slightly tilted to better visualize the ring shaped spn3p-GFP and inner actin distribution (upper inset). In septa, actin (red, Phalloidin, or LifeAct^®^-mCherry labeled) is surrounded by spn3p-GFP (green), confirming that the latter mostly locates closer to the cell periphery, forming a ring-like structure.

**Fig. 4.**
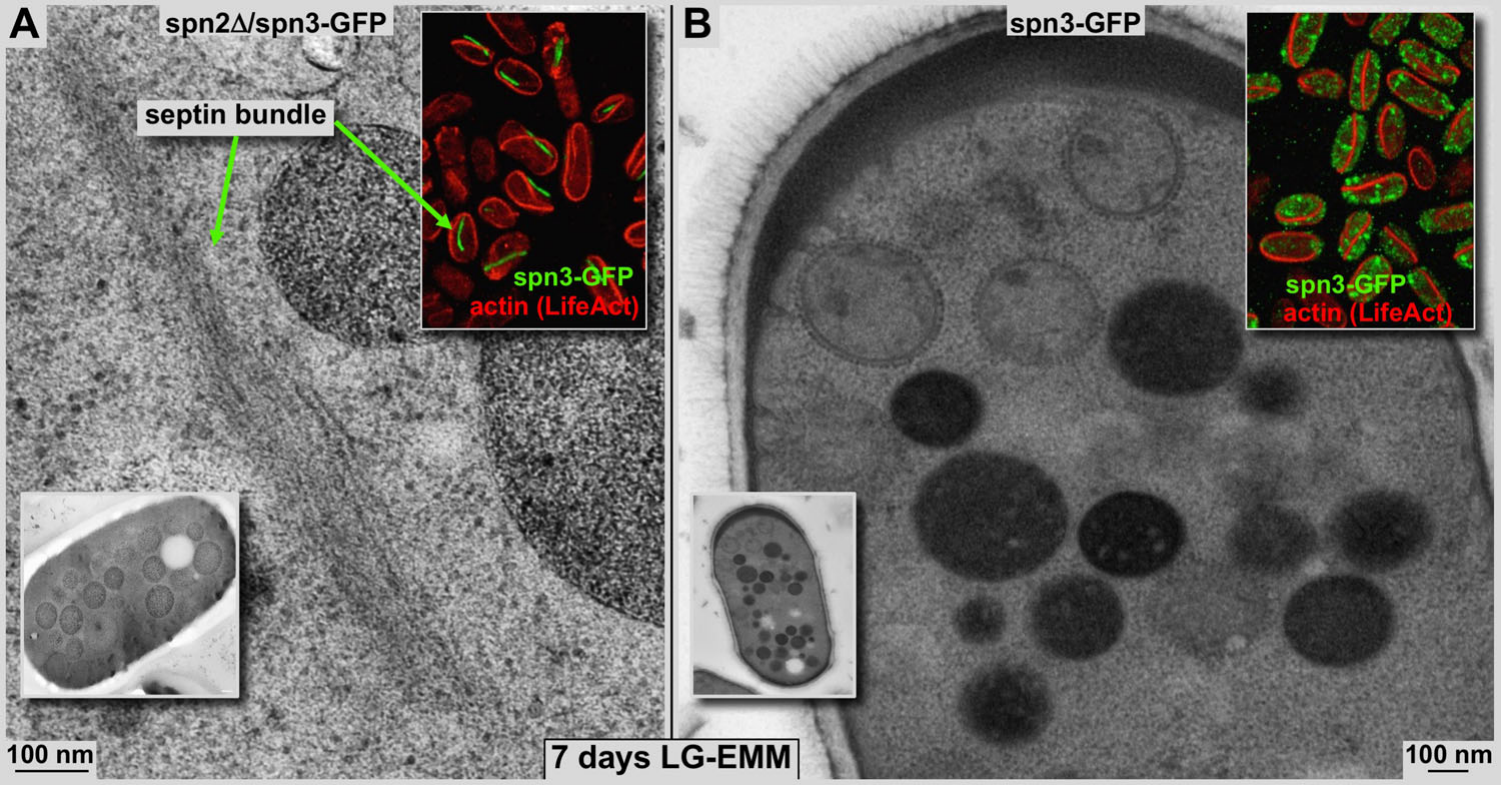
**Filamentous septin assemblies in plastic sections.** Comparison of 80 nm plastic sections of high-pressure frozen, freeze-substituted and plastic-embedded starved spn2Δ/spn3-GFP cells (A) and spn3-GFP cells (B). Both cells were starved for 7 days in low glucose medium as described. The upper inset panels show the corresponding spn3p-GFP fluorescence (green) as well as LifeAct^®^-mCherry, which marks F-actin (also see Fig. 3). In starvation, F-actin forms long filamentous structures in both cell types. The lower inset panels show EM overviews of cells at corresponding conditions. (A) In spn2Δ/spn3-GFP cells, the F-actin bundles and filamentous spn3p-GFP assemblies do not overlap (upper inset panel). Green arrows point to comparable septin bundles on electron micrograph and fluorescence images. (B) None of the filamentous spn3p-GFP assemblies were present in starved wild-type cells even though F-actin forms the same type of bundles as in spn2Δ cells (upper inset panel). Otherwise, the cytosol of both cell types look very similar. Note the dense vacuoles and the lighter stained fragmented mitochondria, which are densely decorated with ribosomes.

**Fig. 5.**
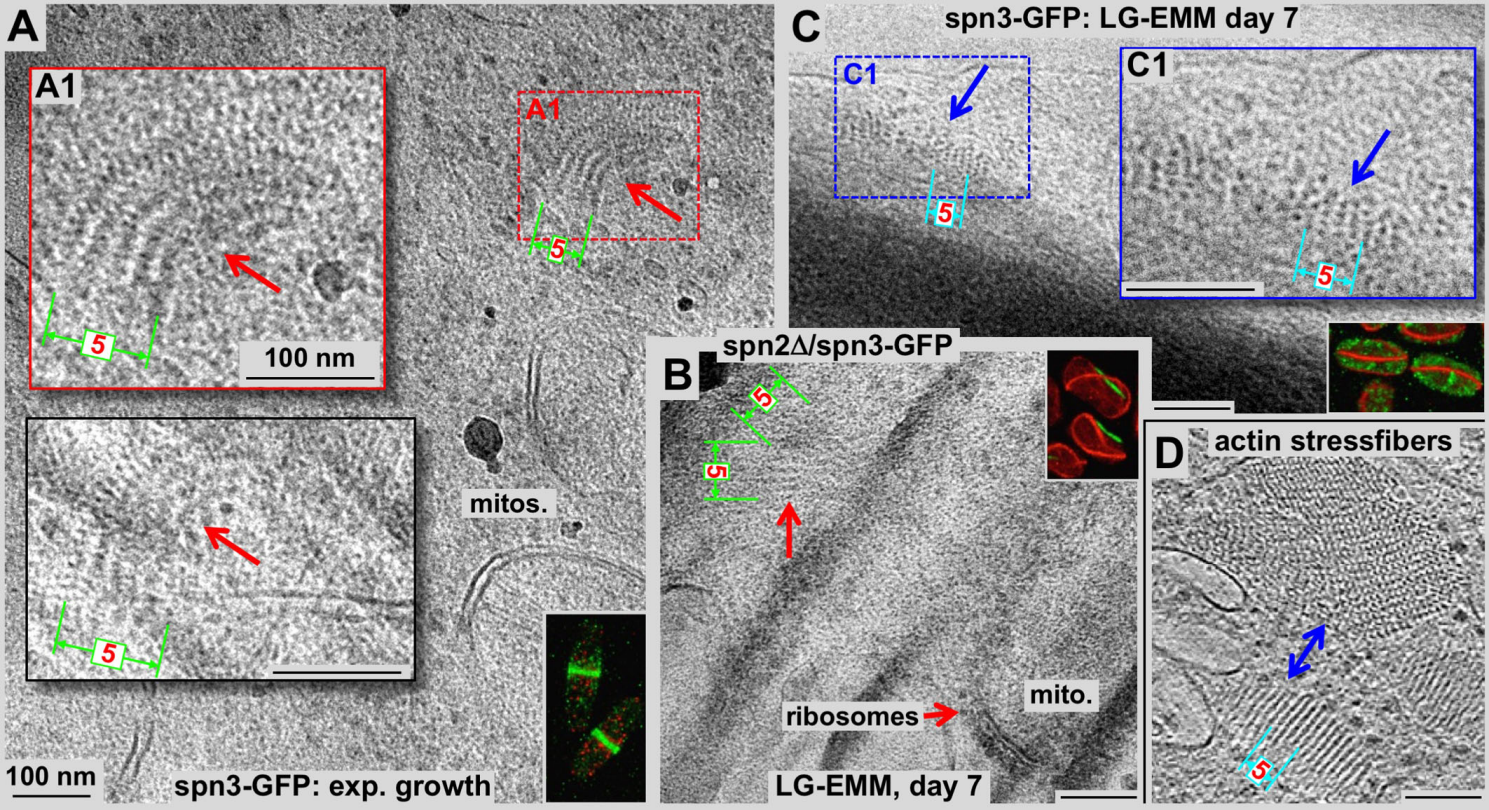
**Septin and F-actin bundles observed in sections of unstained, frozen-hydrated specimens.** (A–C) We have taken advantage of the superb molecular structure preservation of vitrified sections to compare the morphology of F-actin bundles with that of spn3p-GFP aggregates in wild-type (A, exponentially growing; C, starved) and *spn2*Δ cells, and after 7 days of glucose starvation (B). C shows a starved wild-type cell that shows F-actin bundles, but no septin assemblies (also see Fig. 4B). D shows a vitrified section through the stress fibers of a 3T3 fibroblast, allowing for a direct comparison of shape and dimensions with the F-actin bundles in C. The excellent molecular preservation in frozen-hydrated specimens reveals distinct differences between septin (A, including insets; B) and actin bundles (C) or actin stress fibers (D). Both F-actin bundles and stress fibers are morphologically quite different from spn3p-GFP assemblies, which form tighter curves and show a fine, but well visible, repetitive pattern of globular domains while appearing less ordered at the overall bundle level, especially in the clusters found in exponentially growing state (A). The lateral packing of F-actin bundles is much tighter than that of septin bundles [compare the width of five strands within actin bundles (visible in panels C and D, indicated in blue) and septin bundles (panels A and B, indicated in green)]. Insets in A, B and C show corresponding cells with fluorescence labeling of septin-3 (spn3p-GFP) and actin (LifeAct^®^-mCherry).

Here we describe a prominent filamentous spn3p assembly that formed in glucose-starved cells carrying a deletion of the spn2 gene (spn2Δ). A more detailed physiological and biophysical study on these processes is currently in preparation (Heimlicher et al., in preparation), but here we focus on a particular septin-related observation we initially made by electron microscopy. Filamentous spn3p assemblies were identified in electron microscopy pictures with correlative light and electron microscopy (CLEM) (Fig. 2) and with immunolabeling. The structural appearance of the spn3p-GFP assemblies suggests that they represent assembled spn3p filaments. Most likely, these filaments contain spn1p as well, which forms similar assemblies in glucose-starved spn2Δ cells (An et al., 2004). The filamentous spn3p assemblies we observed are different in structure from the metabolic enzyme polymers as reported previously for glucose-starved *S. cerevisiae* cells (Petrovska et al., 2014). Control experiments designed to test the distribution and macromolecular assembly forms of actin within these strains were performed with LifeAct^®^-mCherry as well as Phalloidin-Rhodamine labeling. Both techniques showed convincingly that septin-GFP polymers do not coincide with actin polymers, or F-actin bundles (see Figs 3–5). Here we are focusing on a comparison of spn3p-GFP in wild-type and spn2Δ mutants, which formed distinct fluorescent structures that were further investigated by electron microscopy (EM), both by tomographic 3D reconstruction on thin-sections of plastic-embedded specimens (Figs. 2–4), as well as on thin-sections of frozen-hydrated, vitrified cells (Fig. 5; reviewed in Hoenger and McIntosh, 2009; Bouchet-Marquis and Hoenger, 2011). Previous studies on glucose starvation revealed polymer accumulations within the cytoplasm of *S. pombe* (Joyner et al., 2016; Munder et al., 2016), but since they were reported to be of different origin, we assume that these were different from the septin bundles we observed here.

**Fig. 6.**
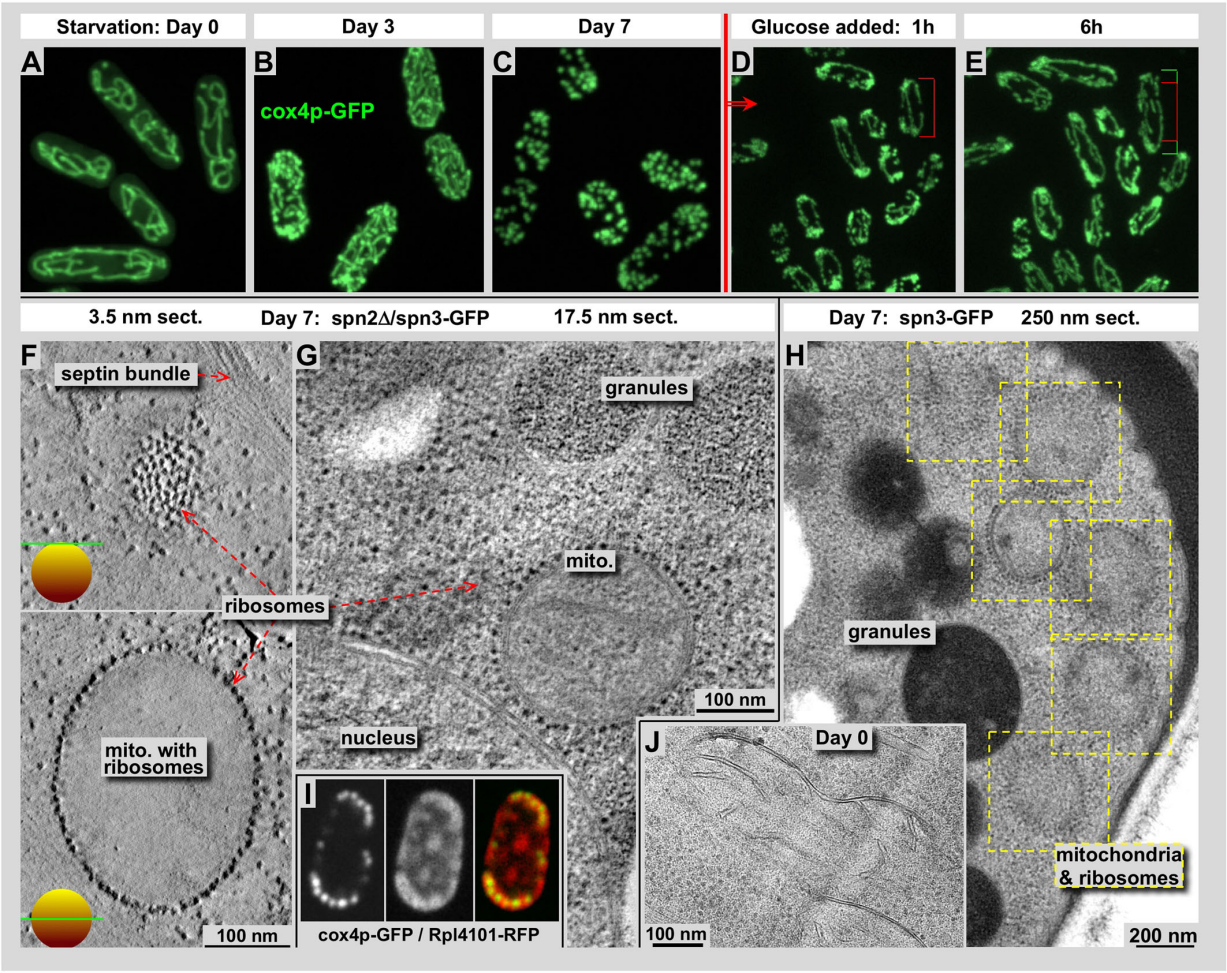
**During prolonged glucose starvation, mitochondria undergo substantial fission and show a massive decoration of their outer membranes by ribosomes.** (A–E) Mitochondria in *S. pombe* at different stages of starvation (A–C) and recovering after adding back glucose (D,E). Mitochondria were visualized with cox4-GFP (Sesaki and Jensen, 1999). A–D show different cells in each panel due to the length of the process. Recovery after adding glucose is much faster than entering starvation, and we could demonstrate how cells start growing and dividing again and elongate their shape gradually (see cell-length markers in D and E, which point to identical cells. The green bar indicates the growth during that time.) EM pictures (F–I) were either tomographic X-Y slices of 250 nm plastic sections (F,G) at 3.5 nm (F) and 17.5 nm thickness (G), a full 250 nm plastic section (H) or a tomographic slice from a cryo-electron tomogram of a vitrified section (J). Panels F–H show cells after 7 days of starvation, while panel J functions as a control of cells during exponential growth. Starved cells show mitochondria outer membranes which are densely packed with ribosomes (F–H), while the membranes of exponentially growing cells are smooth (J). (I) Co-localization of mitochondria, labeled with cox4p-GFP (left), ribosomes labeled with Rpl4101-RFP (center) and the overlay of both (right).

*S. pombe* cells respond to glucose starvation with a complete halt in cell growth and division, as well as a series of large-scale cytosolic changes. Among others, there are distinct modifications of mitochondria length and surface structure. Here we describe our observation from *S. pombe* cells after glucose depletion for 7 days, which reveals substantial mitochondria fission and decoration of their outer membrane by densely packed ribosomes. Mitochondria adopt an almost spherical shape with an average diameter of approximately 300–400 nm, clearly visible by light and electron microscopy techniques. Electron microscopy and tomographic 3D reconstruction of plastic-embedded specimens, sectioned to about 300 nm thickness, reveal a dense, almost crystalline, packing of ribosomes to the outer surface of the strongly shortened mitochondria (see Fig. 6).

## RESULTS AND DISCUSSION

### Distribution of spn3p-GFP and spn1p-RFP in wild-type cells

We induced glucose starvation to *S. pombe* by culturing cells in low-glucose Edinburgh Minimal Media (LG-EMM: see Materials and Methods). The cultures stopped growing within about 2 days of glucose starvation but sub-cellular reorganizations proceeded up to day 7 of culturing (Fig. 1B1–B3,D1–D2). After that, cells remained static or started to die. Therefore, we focused our analysis on cells up to 7 days of starvation (see Materials and Methods, Makushok et al., 2016). All strains are listed in Table 1.

**Table 1.**
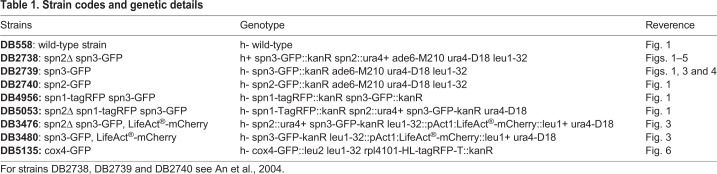
**Strain codes and genetic details**

**Fig. 7.**
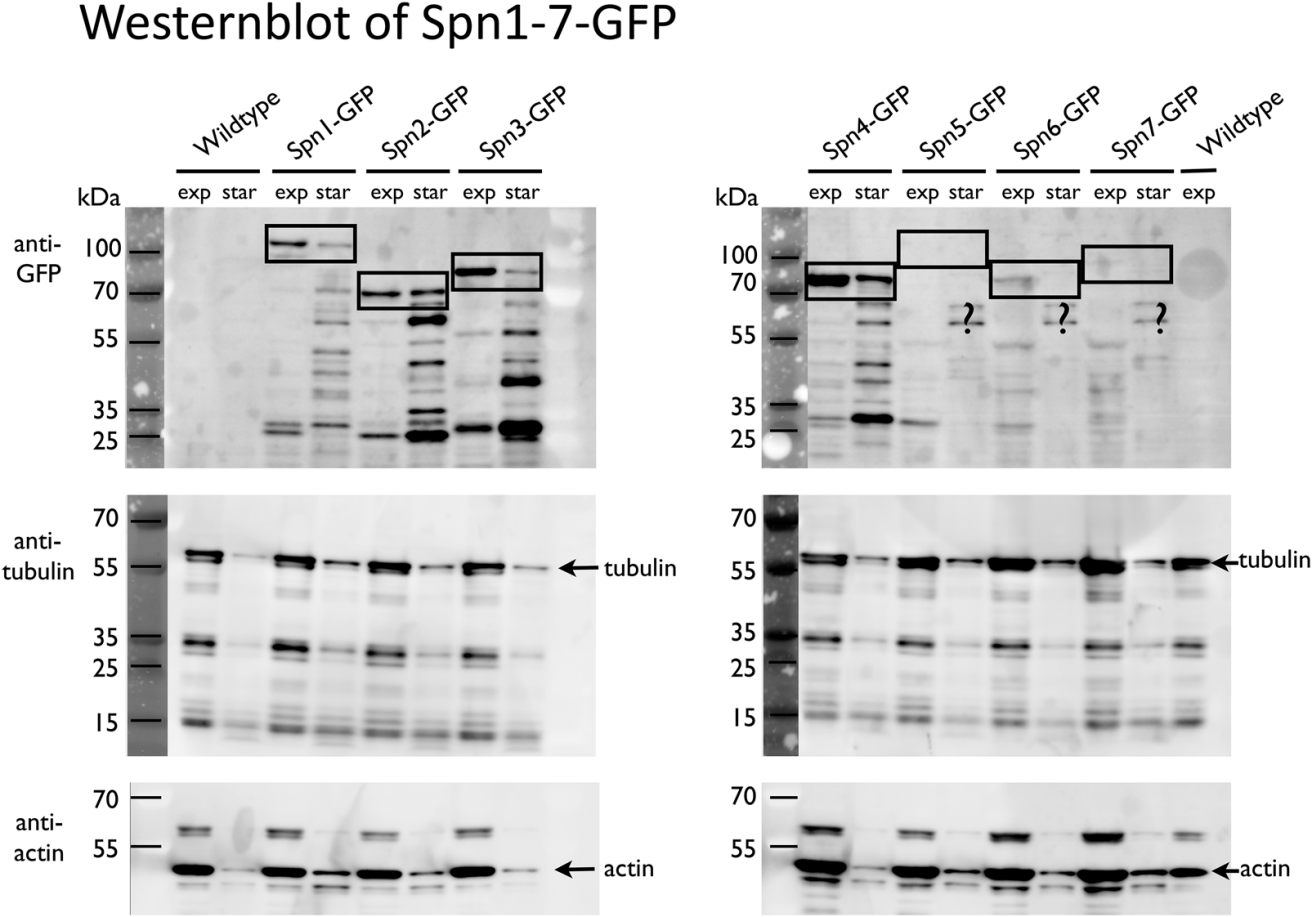
**Western blot with anti-GFP antibodies marking septin-GFP constructs (spn1-GFP to spn7-GFP) and, as control, wild type during the exponential growing phase and after 6** **days of starvation.** Septins 5–7 are expressed mostly during meiosis, and therefore are relatively sparse, especially in non-dividing, starved cell cultures. Septins 1–4 are strongly expressed under both conditions – exponential growth (EG) and after 6 days of starvation (SD6) – but seem to fragment much more during starvation, most likely caused by a tuned-down expression of fresh protein. Tubulin and actin expressions are significantly reduced after 6 days of starvation.

In our first set of experiments, we analyzed the structure and dynamics of spn3p-GFP and spn1p-RFP in exponentially growing [Fig. 1A1–B3 (spn2Δ)], and glucose starved cells [Fig. 1C1–D2 (spn2Δ)] by confocal fluorescence, and differential interference contrast (DIC) microscopy (Fig. 1). In addition, we performed western blotting to show the expression levels of all septins present (septins 1–7) both in wild type and mutants during exponential growth and starvation (Fig. 7). Generally, septins 1–4 are expressed in both conditions, while septins 5–7 are mostly absent. Septins 1–4 seem about equally expressed at both conditions, while septins 1, 3 and 4 appear more fragmented under starvation. Under standard exponential growth conditions cells expressing both spn3p-GFP and spn1p-RFP showed normal growth and division. The expression and cytosolic localization of both tagged proteins was visualized by fluorescence microscopy, shown in Fig. 1A1,A2 and overlaid with a DIC image in Fig. 1A3. We find both spn3p-GFP and spn1p-RFP frequently, but not always co-localizing to multiple tiny clusters scattered throughout the cytosol. In some cells, under specific conditions the proteins formed large assemblies. In dividing cells, both proteins localized to the typical location at the cell periphery where the septin ring forms. At the same time the cytosolic signal decreased, suggesting a reduced cytosolic concentration (Fig. 1A1–A3).

Seven days of glucose starvation completely abolished cell division or growth and reduced the distribution of most of the spn3p-GFP (Fig. 1C1) and spn1p-RFP (Fig. 1C2) to one single aggregate that seems to contain both proteins, though sometimes at seemingly different concentrations (see overlay in Fig. 1C3). However, residual amounts of both proteins can still be detected in the background, but any precise quantitative analyses by the fluorescence microscopy methods used here are not reliable enough for these kinds of interpretations.

### Distribution of spn3p-GFP and spn1p-RFP in cells with a spn2Δ genotype

The overall distribution and accumulation of spn3p-GFP and spn1p-RFP expressing cells was very different when the spn2 gene was deleted (spn2Δ; Fig. 1B1–D3). At exponential growth conditions (Fig. 1B1–B3), spn3p-GFP was mostly found in small aggregates, and at the septa of dividing cells (arrows in Fig. 1B1). Unlike spn3p-GFP, and spn1p-RFP cells, cells with an spn2Δ genotype do not seem to incorporate spn1p-RFP into their septa, but divide and grow normally, and still accumulate spn3p-GFP at their septa [arrows in Fig. 1B1–B3 (overlay with DIC)]. This differs from earlier findings based on GFP-tagged spn1p, which formed a ring during cytokinesis in the absence of spn2p. Furthermore, this result challenges the previous hypothesis, which states that spn1p is essential for the other septins to form a ring (Wu et al., 2010).

Similar to wild-type cells, 2–3 days of culturing spn2Δ cells results in complete growth arrest. Fig. 1D1–D3 shows spn2Δ cells and the distribution of spn3ps-GFP and spn1p-RFP after 7 days of starvation. Interestingly, in such starved cells both spn3p-GFP and spn1p-RFP co-localized into what appears to be large, straight filamentous assemblies, or bundles, consuming almost all protein and leaving only trace amounts still visible throughout the cytosol. Consistently, each cell contained only one single filamentous assembly (Fig. 1F). However, not all of these filamentous spn1p-RFP assemblies contain spn3p-GFP (arrows in Fig. 1D1–D3). Hence, it appears as if spn3p can only form filaments together with spn1p, but spn1p may form filaments in the absence of spn3p. These assemblies were also very well visible by EM and electron tomography (ET), which initiated a brief study of spn3p-GFP assembly morphology that served to establish a correlative light and electron microscopy approach (see Fig. 2).

### Correlating septin bundles from light to electron microscopy

Today, fluorescence light microscopy (LM) in its multiple forms, employs super-resolution methods and genetically encoded fluorophores such as GFP and mCherry, allowing imaging of large macromolecular complexes and organelles within a cellular context and in a fully hydrated *in vivo* state (reviewed in Li et al., 2015). This is obviously a significant advantage over EM. Despite some recent efforts in viewing hydrated biological specimens in a fluid chamber by EM (reviewed in Evans and Browning, 2013), examination of active living cells in the electron microscope is still very difficult and far from a routine application. Nevertheless, despite spectacular progress in super-resolution light microscopy, detailed structural investigations *in vitro* and *in situ* on macromolecular complexes and sub-components thereof are still the domain of EM simply because EMs feature superior resolution and independence of labeling tools, which, however, could be seen as both a blessing or a curse. The absence of anything comparable to fluorescent labels sometimes interferes with an unambiguous identification of structures, especially in the crowded environment of an intact cell depending on the questions asked.

Since the detectable signals with fluorescence-based microscopy mostly come from distinct proteins labeled with fluorochromes, there is little else that obscures the images. In contrast, electron microscopy, in particular of unstained, frozen hydrated specimens, typically produces 2D projections that includes all electron scattering structures such as proteins, lipids, glycosylation and whatever else is there, often with very low contrast (Dubochet et al., 1988; Hsieh et al., 2002; Al-Amoudi et al., 2004). Hence, while fluorescence LM is ideal for the identification of specifically labeled targets, in the EM we have to deal with large arrays of superimposed densities and most of them cannot be directly identified. On the other hand, EM reveals these structures with finer molecular detail and within their larger context, which is usually not visible by fluorescence microscopy due to the limits of how many structures can be fluorescently labeled within the same preparation. Addressing these issues, CLEM methods are in great demand and by now have produced some exciting results (e.g. see Kolotuev et al., 2010; Kukulski et al., 2011).

To verify the nature of the filamentous septin assemblies we have observed by EM, we engaged in a straightforward CLEM approach. To this end, we correlated four different levels of microscopy: a phase contrast image (Fig. 2A), a fluorescence image (Fig. 2B), an overview EM micrograph (Fig. 2C) and a thin computational slice through a tomographic 3D reconstruction (Fig. 2D) – all recorded from the very same specimen. Using this CLEM method, we investigated the spn3p-GFP containing septin assemblies in intact starved *spn2*Δ cells in a 250 nm thick plastic section (Fig. 2A–C), as well as a tomographic 3D reconstruction thereof (Fig. 2D). We achieved an unambiguous correlation of the spn3p-GFP fluorescence with the dense assemblies observed in plastic or vitrified sections. In the figures, blue arrows connect identical elements such as entire cell outlines, or the large piece of dense and highly fluorescent material. Likewise, two dense granules seen by electron microscopy in Fig. 2C are connected by blue arrows to the 40 nm tomographic slice in Fig. 2D. The filamentous spn3p-GFP containing assembly itself (connected by green arrows) is not visible by phase contrast, but shows up well by fluorescence microscopy and can be seen as an elongated density in the overview electron micrograph (Fig. 2C). Finally, our analysis reveals more molecular details about the assembly's filamentous supra-molecular nature within a thin slice of the tomographic 3D reconstruction (Fig. 2D). Seven days of glucose starvation significantly enhanced spn3p-GFP bundling in spn2Δ mutants (Figs 1D1–D3 and 3A) as compared to the wild type (Figs 1C1–C3 and 5A). These filamentous assemblies appeared to be quite straight (Fig. 2D).

### Immunolabeling of spn3p-GFP by anti-GFP primary and gold-linked secondary antibodies

To further confirm the presence of spn3p-GFP at a higher spatial resolution within the septa and filamentous assemblies that we had observed by EM in spn2Δ/spn3-GFP cells (Fig. 2), we employed immunogold labeling using a 15 nm gold-linked antibody system directed towards GFP (Fig. 3). Cellular preparations embedded in plastic often preserve antigens of epitopes such as the GFP tag used here. Hence, when these epitopes are accessible at the surface of a section they can be decorated by antibodies and observed in both light as well as electron microscopes (reviewed in Griffiths and Lucocq, 2014). Immunolabeling against GFP domains confirmed the presence of the spn3p-GFP protein within the dense filamentous assemblies formed in spn2Δ spn3p-GFP cells after 7 days of glucose starvation (see Fig. 3A, both examples are recorded at identical conditions). The insets show fluorescence labeling of actin and septin-3 with LifeAct-mCherry (upper inset).

In a control experiment, we examined septa in exponentially growing cells with anti-GFP immunolabeling (there are no septa anymore in starved cells). In spn3p-GFP expressing cells we could detect the presence of GFP at the site of cytokinesis by EM, and consequently that of spn3p-GFP in 100 nm thick plastic sections (Fig. 3B). Fluorescence microscopy revealed a very obvious, dense GFP signal within the septa (Fig. 3B insets), and on plastic sections GFP-antibody immunolabels find spn3p-GFP there as well. In both panels of Fig. 3, the lower fluorescence insets show projections of a thin slice through a confocal stack with actin labeled with Rhodamine-Phalloidin, overlaid with a phase-contrast image. The upper insets show projections through a full confocal 3D stack that are slightly tilted and shows actin labeled with LifeAct-mCherry. The tilting reveals dividing cells and their septa (Fig. 3B) from an oblique angle. This data illustrates that most of the spn3p-GFP (green) locate to the outer periphery of the septum, with some actin (red) mostly towards the center, which is in accordance with the immunogold labeling of spn3p-GFP (Fig. 3B).

### Septin–actin interactions

To further clarify the nature of the spn3p-GFP assemblies we tested whether actin may play a role in their formation. We visualized actin either with Rhodamine-Phalloidin (Fig. 3A, lower inset) or LifeAct^®^-mCherry (Figs 3–5) to make sure actin cable formation was not triggered artificially by any of these stains. The Phalloidin-stained images (Fig. 3A, lower inset) are thin confocal sections, while the cells stained with LifeAct^®^-mCherry (Figs 3A upper inset, 4 and 5) are entire 3D stacks that also allowed us to rotate them for a better view of septa (Fig. 3B). Both methods revealed clear, identical signals (Riedl et al., 2008). Here we found that glucose starvation of *S. pombe* cells, independent of the presence or absence of septin-2, not only modifies septin aggregates, but also triggers the formation of long actin cables (inset panels in Figs 3A and 4). Most importantly, under any condition, actin and spn3p-GFP do not co-localize, no matter whether spn3p-GFP accumulates into filamentous assemblies as found within spn2Δ cells (insets in Figs 3A and 4A), or remain randomly distributed within small clusters of spn3-GFP expressing cells (insets in Figs 3B and 4B).

### Morphological differences between septin assemblies and actin bundles observed in vitrified sections

In this study, we expanded our EM imaging with preparations of vitrified sections from frozen-hydrated cellular samples to avoid potential artifacts from chemical fixation or freeze-substitution (see Fig. 5). As of today, most ultra-structural research into intact cells is still carried out by at least some mild chemical fixation during freeze-substitution and embedding in plastic for cutting thin sections by a microtome. Due to effects of chemical fixation and/or freeze-substitution this technique produces image data of cellular structures to an interpretable resolution of approximately 4–5 nm, and rarely below (e.g. see Kellenberger, 1991; McDonald et al., 2010). For molecular data beyond that samples have to be imaged in a frozen-hydrated, vitrified state. For EM studies vitrification is the only preparation method that preserves bio-molecular structures to near-atomic detail. While vitrification is a common process for structural investigations on isolated macromolecular complexes, it is still a rather tricky process to be applied to cellular specimens (e.g. see Bouchet-Marquis and Hoenger, 2011; Dubochet et al., 2007). Attempts to create frozen-hydrated cellular preparations for electron microscopy date back to pioneering work by Christensen, 1971, and later McDowall et al., 1983. However, cryo-EM as we know it today was not yet invented, or was just about in its very early stages of development (Dubochet et al., 1988), and the real breakthrough for vitrified sections came much later with work from Hsieh et al., 2002 and Al-Amoudi et al., 2004.

To further strengthen the data that we have obtained from plastic sections, we also prepared vitrified sections from intact spn2Δ cells during exponential growth (Fig. 5A) and after 7 days of glucose starvation (Fig. 5B,C). As explained above, the rationale for using vitrified sectioning was to obtain sufficient resolution and unobstructed molecular detail that would allow us to directly distinguish F-actin bundles from filamentous spn3p-GFP assemblies by their distinct morphologies, without having to worry about chemical fixation and/or dehydration artifacts. Interestingly, we could not detect any structure convincingly showing F-actin bundles in any of the glucose-starved cells, although the strong fluorescence signal suggested the presence of 1–2 prominent F-actin cables that should be visible in any of the sections (Fig. 4). It is therefore possible that the architecture of these starvation-specific actin cables that we found in our preparations (Fig. 5C) differ considerably from that of F-actin stress fibers (Fig. 5D). To illustrate the different morphologies of F-actin bundles and filamentous spn3p-GFP assemblies we resorted to a picture of vitrified, cultured 3T3 fibroblast cells as shown in Fig. 5D (also see Bouchet-Marquis and Hoenger, 2011), acting as a control for actin bundle dimensions and packing. This vitrified section reveals projections at various angles through actin stress fibers (blue arrows in Fig. 5D). These clearly show a very different morphology when compared to the filamentous spn3p-GFP assemblies we had identified. Similarly, in exponentially growing cells the much shorter, curvier and less abundant spn3p-GFP structures showed a characteristic morphology that differed substantially from actin filament arrangements such as those found in stress fibers (Fig. 5D). The pattern of the filamentous spn3p-GFP assemblies (Fig. 5 red frame with a blow-up in the inset panel) often resembled a tightly pitched staggered assembly, which lack the long-pitched, helical pattern and stiffness of actin filaments (e.g. see Bremer et al., 1991). All four panels of Fig. 5 are at the same magnification (see scale bars), and by comparing these images actin bundles appear significantly narrower and more tightly packed than filamentous spn3p-GFP assemblies. In addition, filamentous spn3p-GFP assemblies or clusters appear less ordered. Nevertheless, assuming that the overall structure of F-actin should still be the same despite some differences in arrangement, the bundles in Fig. 5C show an F-actin packing arrangement that is very different from what we find in filamentous spn3p-GFP assemblies or clusters (Fig. 5A,B). On the other hand, the pattern of the spn3p-GFP assemblies in our images match quite well the structure and dimensions of the EM 3D reconstructions that have been presented by other groups (Lukoyanova et al., 2008).

### Mitochondria decoration by ribosomes upon glucose starvation

During our EM analyses of glucose-starved *S. pombe* cells we discovered an interesting side-effect of starvation on mitochondria: they undergo substantial fission (Fig. 6A–C), and their outer membrane becomes decorated with ribosomes (Fig. 6F–J). This effect is visible by EM in both spn-3-GFP cells (Fig. 6A–E,H,J) and spn2Δ/spn-3-GFP cells (Fig. 6F,G), and was indistinguishable between the two genotypic types. After about 3–4 days of starvation mitochondria begin massive fission and transfer from the commonly observed tubular shape to small spheres (Fig. 6A–C). Upon addition of glucose to the starving cells, this condition is quickly reversed (Fig. 6D,E) and cells start growing again (see red and green cell-length markers in Fig. 6D,E). In addition, after several days of glucose starvation their outer membrane surface is fully decorated with ribosomes, as confirmed by tomographic slices through the spheres (Fig. 6F,G). Fig. 6I shows an overlay of the ribosomal marker rpl4101, tagged with RFP and the mitochondrial marker cox4p-GFP (strain 5135). While ribosomes are widely distributed throughout the cytosol, they clearly accumulate in close proximity to mitochondria. The nucleus sometimes shows some decoration as well, but this could be caused simply by ribosomes originating from the continuation of the nuclear membrane into rough endoplasmic reticulum (RER). The mitochondrial outer membrane is not connected with RER and features different properties such as large amounts of voltage gated anion channels (VDAC; Dubey et al., 2016) or associated proteins [e.g. hexokinase (HxKII; Colombini, 1983)] that could be a potential anchor points for the ribosome association observed here. The packing of ribosomes to the outer mitochondria membrane is tighter than what one typically finds on rough endoplasmic reticulum. Both modifications, mitochondria fission and ribosome decoration, are quickly reversed upon glucose addition, which restarts cell growth and division to normal levels within hours. Addition of glucose reinstates long tubular mitochondria with smooth surfaces that we find at exponential growth conditions (Fig. 6J). Other organelles with membranes such as the vacuoles do not show any ribosome decoration. We have not found any reports in the literature on comparable observations, neither on yeast nor on other cell lines. Hence, we could only speculate that the ribosomes on the mitochondria surfaces during starvation are mostly inactive. However, upon glucose addition, they optimize the restart of protein synthesis most relevant to ATP production, one of the first things required to get cells back to normality.

### Conclusions

This work combines a technical component with a biological application. On the biology side, we investigated the behavior of septins in *S. pombe* upon extended glucose starvation. Thereby we made some unexpected observations: upon glucose depletion, septin-1 and -3 in *S. pombe* cells with a spn2Δ background aggregate into filamentous assemblies (Figs 1–5). In all types of starved cells explored here, actin forms cables, which do not co-localize with the filamentous septin assemblies or other septin aggregates (Figs 3–5). Regarding functional aspects of septins in *S. pombe*, we could demonstrate that *S. pombe* cells with a spn2Δ background still grow normally (based on observed growth rate and density the cultures reached after the exponential phase), despite aggregating the majority of GFP-labeled septin-3 into small particles throughout the cytosol. Spn3p-GFP is clearly present in septa of wild-type cells (wild type with respect to Spn2p deletion), and spn2Δ/spn3p-GFP mutant cells. However, the septa of spn2Δ/spn3p-GFP mutant cells do not seem to contain any traces of spn1p-RFP anymore. Hence, the overall structure of septa in spn2Δ/spn3p-GFP cells seems to be insensitive to the absence of septin-3 (see Fig. 1) and still function normally. Under wild-type conditions, septin-1, -2, -3 and -4 are expressed in vegetatively growing cells and form hetero-octamer septin rod, which can further assemble into regular septin assemblies (Hall et al., 2009).

F-actin cables in starved *S. pombe* cells (Fig. 5C), mutants or wild type, appear less densely packed than F-actin stress fibers such as in fibroblasts and other motile cells (Fig. 5D, 3T3 cells). There is no indication that any type of myosin motor co-localizes with starvation-induced F-actin bundles. The bundles would be too tight to accommodate myosin motors and cargo within. However, any myosin mediated transport processes could take place on their outer surface.

On the technical side, this work is a CLEM study (Figs 2–4; Kukulski et al., 2011; Schwartz et al., 2007; Briegel et al., 2010), where we investigate the structure of a novel septin protein-assembly that we found in mutant, glucose starved *S. pombe* cells. Our work correlated LM and EM 3D data of a complex protein structure within the cytosol of *S. pombe*. Due to the crowded density of a cytosol, EM alone often may not be sufficient to unambiguously identify such structures, unless they are of very obvious shape and size (e.g. microtubules, ribosomes). Clonable high-density labels for EM, somewhat analogous to GFP, are available but difficult to handle and still part of an emerging technology (Bouchet-Marquis et al., 2012; Morphew et al., 2015).

Here we demonstrate the power of correlative LM and EM for molecular studies in intact cells. By blanking out all unlabeled cellular structures, a fluorescence microscopy image is reduced to one or few fluorescently labeled target structures within a cellular context. This greatly enhances visibility and renders it a highly complementary tool for an unambiguous identification of the structures of interest within the complex density pattern of electron micrographs. We present results on spn3p-GFP in the presence or absence of septin-2 because this combination produced morphologically striking results that could be easily observed by both LM and EM. Wild type or other mutants did not show such an obvious phenotype.

Our results, which we have obtained with vitrified sectioning on frozen-hydrated cells, opened another perspective to septin assemblies and polymers. Omitting staining and/or any type of chemical fixation produces the true protein density of a macromolecular assembly. However, vitrified sectioning is still an emerging technology and will require further refinements, or other means of production. Producing vitrified sections of cellular specimens with a cryo-microtome is difficult and sometimes tedious (e.g. see Bouchet-Marquis and Hoenger, 2011; Dubochet et al., 2007), and correlative approaches are even more difficult because the handling of vitrified material requires uninterrupted cryo-conditions. This means keeping specimens at, or below −140°C and access to specialized tools (Schwartz et al., 2007; Briegel et al., 2010). Also, despite using a newly developed cryo-light microscope in our lab (34, 35) we could not produce enough fluorescence for an unambiguous localization of spn3p-GFP polymers within vitrified sections. Unlike plastic sections of 200–300 nm thickness that we regularly use for tomographic data acquisition (see Fig. 2) our vitrified sections seem to be either too cold and/or too thin (∼80 nm) and therefore do not contain enough active fluorochromes to produce sufficient signal at temperature below −140°C (the vitrification boundary of water). Nevertheless, the accurate structural preservation of biological matter in vitrified sections sometimes allows for a direct *in situ* interpretation and comparison of large macromolecular densities such as the two types of filamentous structures found here. As demonstrated in Fig. 5, examining their morphology in frozen-hydrated preparations was sufficient to assign septin3 to the filamentous polymers that we find in spn2Δ/spn3p-GFP cells with a high probability.

With regard to the dense decoration of mitochondria outer membranes with ribosomes, we do not yet know what exactly the biological functions of these modifications might be. Our educated guess would be that once glucose comes back mitochondria as the primary energy providers should be jump-started as soon as possible for an optimal support of all other metabolic processes.

## MATERIALS AND METHODS

### Cell culture and starvation

*S. pombe* cells (see Table 1 for strains used) were plated by autoclaved toothpicks from frozen glycerol stock for 48 h at 32°C on YE5S (general purpose rich media) plate made from YE5S powder (Sunrise Science Products). Colonies were picked up by autoclaved toothpicks and put in 10 ml homemade EMM supplemented with the relevant extra amino acids in 200 ml flask, shaking at 25°C, 220 rpm for 15∼17 h to 0.4∼0.8 OD. The liquid culture was then diluted to OD 0.05 in fresh EMM and relevant extra amino acids, shaking at 25°C for 13∼15 h to 0.4∼0.8 OD. Subsequently, the culture was diluted to OD 0.05 in fresh EMM [regular (20 g/l glucose) or low (5 g/l glucose)] with the respective extra amino acids and left shaking at 25°C, 220 rpm. All flasks were covered with stainless steel caps without any additional sealing. To analyze cells at an exponential growth phase, cells were harvested about 13∼15 h after the last dilution step. To obtain fully starved conditions, cells were harvested 7 days after the last dilution.

### Homemade EMM

This EMM was made by 3.0 g/l potassium hydrogen phthalate, 2.2 g/l Na_2_HPO_4_, 5.0 g/l NH_4_Cl, 20.0 g/l glucose, 20 ml/l 50× salts stock (52.5 g/l MaCl_2_ • 6H_2_O, 0.735 g/l CaCl_2_ • 2H_2_O, 50.0 g/l KCl and 2.0 g/l Na_2_SO_4_), 1 ml/l 1000× vitamins stock (1.0 g/l pantothenic acid, 10.0 g/l nicotinic acid, 10 g/l inositol and 10 mg/l biotin) and 0.1 ml/l 10,000× minerals stock (5.0 g/l boric acid, 4.0 g/l MnSO_4_, 4.0 g/l ZnSO_4_ • 7H_2_O, 2.0 g/l FeCl_2_ • 6H_2_O, 0.4 g/l molybdic acid, 1.0 g/l KI, 0.4 g/l CuSO_4_ • 5H_2_O and 10.0 g/l citric acid). All the solution stocks were filtered and the media solution was autoclaved at 108°C for 12 min before use.

### Protein tagging and constructs

Protein tagging of the ribosomal protein rpl4101 (Fig. 6I) was performed as described in (Bähler et al., 1998), using the primers: AAGAAGAAAGATGAGAGCTAGATCGTAAGTGTTGTATTATATTACTCAGACCTGCAGACTTCTAACTTTGAATTAGTAAAATCCTTGGAGCTCCTTCAGG and: AGGAAGAGGCCTGCCATGATATCCAAGCGTCACGCTAGGGACCGCTTAATTCAGTCCATCGAGCTGTAGATGATCCGTGAATTCGAGCTCGTTTAAAC.

The plasmid TagRFP-T was amplified from the Addgene plasmid #44906 (Lee et al., 2013) with primers: ATCCTTGGAGCTCCTTC and ATCGGATCCCCTTATACAATTCATCCATACC and inserted with restriction enzymes PacI/BamHI into the tagging plasmid equipped with a ‘happy linker’ (HL) sequence between gene and fluorophore sequence as for EB1 in (Jankovics and Brunner, 2006).

### Light microscopy

Glass slides were discharged by Emitech K100X Glow Discharge (Emitech, Fall River, USA) then covered with 8 μl Lectin from *Bandeiraea Simplicifolia* solution (2 mg/ml) (Sigma-Aldrich) and air-dried. A drop of yeast culture was placed on the slides and after 10 min incubation was rinsed with culture supernatant. Observations were made at 25°C under Nikon Plan Fluor 100× oil lens (Nikon, Japan) by Nikon Eclipse 80i (Nikon, Japan) and Zeiss 510 Laser Scanning Confocal Microscope (Zeiss, Germany). Data was collected and processed by NIS-Elements AR 3.2 for Nikon Eclipse 80i and ZEN 2009 for Zeiss 510. Actin was fluorescently stained with LifeAct^®^-MCherry (Riedl et al., 2008) or with Rhodamine-Phalloidin (Molecular Probes/Thermo Fisher Scientific). Mitochondria in Fig. 6 were labeled with cox4p-GFP (Sesaki and Jensen, 1999). Ribosomes in Fig. 6I were marked with Rpl4101-RFP (Jankovics and Brunner, 2006).

### Electron microscopy and tomography

*Schizosaccharomyces pombe* cells were directly high pressure frozen as a solution, while the fibroblast cells of Fig. 5D were plated onto carbon-coated sapphire discs as previously described (reviewed in Dubochet et al., 2007). Both specimens were high pressure frozen using a Wohlwend Compact-2 high-pressure freezer (Martin Wohlwend AG, Sennwald Switzerland). *S. pombe* samples destined for plastic section microtomy were freeze-substituted in 0.1% glutaraldehyde and 1% uranyl acetate in acetone for 48 h and warmed from −90°C to −50°C in 8 h (5°C per hour). Cells were then washed by acetone three times and infiltrated in HM20 solution (25%, 33%, 50%, 67%, 75%, 100% in acetone) (Lowicryl HM20 Embedding Kit, Electron Microscopy Science, Hatfield, USA) over 5 days using Leica EMAFS (Leica, Vienna, Austria). Samples were then polymerized to blocks under Leica EMAFS UV light unit for 72 h.

Plastic blocks were cut into ribbons of 80 (for single projection images) to 250 nm thick plastic sections (for tomographic volume reconstructions), depending on the questions asked, by Leica Ultracut microtome (Leica Inc., Vienna, Austria) using Diatome Ultra 45° (Diatome AG, Biel, Switzerland). For electron tomography, (Figs 2D, 4A and 5B,C) the thickness of section was around 250 nm. For immunolabeling, the thickness of a section was around 80 nm (Fig. 3). Ribbons were collected on formvar-coated Cu-Rn grids (Electron Microscopy Science) or Carbon Film Finder grids (Electron Microscopy Science), immunolabeled (optional), stained by uranyl acetate (2% uranyl acetate in 70% methanol) for ∼4 min and Reynold's lead citrate for ∼2 min (the staining time was adjusted based on the thickness of the sections).

The immunolabeling process was done in a humid chamber to prevent evaporation of the solvents. Sample grids were blocked in 1% non-fat dry milk in PBST (Phosphate Buffered Saline, Tween-20) for 30 min. Primary antibody (homemade poly-clonal Rabbit IgG anti GFP, a generous gift from Pearson Lab, University of Colorado, Denver) was diluted in blocking buffer as 1:100. Second antibody (EM Goat anti-Rabbit IgG 15 nm Gold, Ted Pella, Redding, USA), was diluted 1:20 in blocking buffer. Grids were placed on the drop of primary antibody solution for 2 h. Grids were rinsed three times with PBST and then placed on the drop of secondary antibody solution for 1 h. Grids were again rinsed with PBST three times and then with distilled water another three times. Grids were finally dried by air at room temperature.

Single pictures of immunolabeled sections were acquired with a FEI Philips CM100 TEM and AMT 2Kx2K bottom-mount digital camera. Dual-axis serial section montaged tomograms were acquired with a Tecnai TF30 300 kV FEG (Thermo Fisher Scientific) transmission electron microscope using SerialEM (Mastronarde, 2005) from ±60° with 1° increments, recorded with a 4K GATAN Ultrascan-895 CCD camera (GATAN Inc. Pleasanton, USA). R-weighted back projection tomograms were computed using IMOD (Kremer et al., 1996).

2D projections and tomograms of vitrified sections (Fig. 5) were obtained by a similar procedure as described above, but with some obvious adaptations to the nature of frozen-hydrated specimens, following modified protocols originally described in Al-Amoudi et al., 2004 (also see Bouchet-Marquis & Hoenger, 2011). Essentially, intact cells were high-pressure frozen as described above, but with the freeze-substitution step omitted, the frozen blocks were directly sectioned with a cryo-ultra microtome into ∼80 nm thick vitrified sections (about the maximum thickness reasonably achievable by this technique). Grids with vitreous cytoskeletons were mounted on a GATAN-626 cryo-holder and imaged in a Tecnai TF30. Images were obtained through a Tridiem Gatan Imaging Filter operating with a slit width of 20 eV around the zero-loss range and recorded onto the Ultracam-868, a prototype 4K lens-coupled CCD camera (Gatan Inc., Pleasanton, USA). A typical tilt-series ranged from ±60° tilt angles with 2° tilting increments and a pixel size of 0.776 nm^2^. The imaging defocus was either −6 μm or −4 μm to produce sufficient phase contrast. Tomograms were calculated with R-weighted back projection using IMOD, the contrast transfer function (CTF) was corrected (Xiong et al., 2009) and the strong, spread-out signals of the gold fiducial markers were computationally erased.
